# Evaluation of CD44s, CD44v6, CXCR2, CXCL1, and IL-1β in Benign and Malignant Tumors of Salivary Glands

**DOI:** 10.3390/diagnostics12051275

**Published:** 2022-05-20

**Authors:** Fonthip Laohavisudhi, Titikorn Chunchai, Natnicha Ketchaikosol, Wacharaporn Thosaporn, Nipon Chattipakorn, Siriporn C. Chattipakorn

**Affiliations:** 1 Department of Oral Biology and Oral Diagnostic Sciences, Faculty of Dentistry, Chiang Mai University, Chiang Mai 50200, Thailand; fonthip_lao@cmu.ac.th (F.L.); natnicha.ketc@cmu.ac.th (N.K.); wacharaporn.th@cmu.ac.th (W.T.); 2 Cardiac Electrophysiology Research and Training Center, Faculty of Medicine, Chiang Mai University, Chiang Mai 50200, Thailand; titikorn.c@cmu.ac.th (T.C.); nipon.chat@cmu.ac.th (N.C.); 3 Center of Excellence in Cardiac Electrophysiology Research, Chiang Mai University, Chiang Mai 50200, Thailand

**Keywords:** CD44s, CD44v6, CXCR2, IL-1β, salivary gland tumor

## Abstract

Background: Several studies have reported an association between high expression of CD44 in different types of cancer. However, no study has reported a link among CD44 expression, other biomarkers, and the aggressiveness of salivary gland tumors. Methods: A total of 38 specimens were obtained from non-tumorous salivary glands, benign and malignant tumors in salivary glands. Immunohistochemical analyses of CD44s, CD44v6, IL-1β, CXCL1, and CXCR2 were performed, and the area of positive cells was assessed. Results: We found that both CD44s and CXCR2 expression were increased in the benign and malignant groups. CD44v6 was also increased in both groups, but it had the highest level in the malignant group. IL-1β was the only biomarker that increased significantly in the malignant group in comparison to the other two groups. Conclusions: CD44s, CD44v6, CXCR2, and IL-1β expressions were found to be higher in salivary gland tumors. However, IL-1β alone may play a crucial role in the aggressiveness of salivary gland tumors as this cytokine was expressed only in the malignant group with high expression associated with high-grade malignancy.

## 1. Introduction

In each year, 0.4 to 13.5 people per 100,000 have been diagnosed with salivary gland tumors [1,2]. Pleomorphic adenoma is the most common benign type of salivary gland tumor, while mucoepidermoid is the most common type of malignant salivary gland tumor [3,4]. Salivary gland tumors have complexity in histopathological features with various clinical characteristics. Several biomarkers, including aldehyde dehydrogenase (ALDH), cluster of differentiation 44 (CD44), and octamer-binding transcription factor (OCT4), have been used to identify tumor stem cells in various tumors, including, tongue squamous cell carcinoma and major and minor salivary gland neoplasms [5,6].

CD44 is essential for cell adhesion, interaction, and migration [7]. There are variant isoforms of CD44, such as CD44s and CD44v6 [7]. Previous studies have reported a high expression of CD44s in several types of cancers, including pancreatic, colon, and breast cancers, as well as lymphoma [8,9,10]. In addition, CD44v6 expression has been linked to a poor prognosis and metastasis of breast cancer [11]. However, the association between CD44s and CD44v6 expression in benign and malignant of salivary gland tumors has not been studied. In addition to CD44, an increase in pro-inflammatory cytokines, including IL-1β, CXCL1, Nuclear Factor Kappa B (NF-κB), and their downstream signaling pathways, have been found to be associated with oral squamous cell carcinoma [12,13,14]. Inflammation, apoptosis, and host resistance are mostly mediated by cytokines, which also act as signaling molecules in cell–cell interactions [15]. A recent study also reported that interleukin-6 (IL-6) and interleukin-8 (IL-8) are candidate markers for oral squamous cell carcinoma [16]. A growing body of research shows that increased CD44s, CD44v6, IL-1β, CXCL1, and CXCR2 expressions have been associated with different kinds of tumors [12,17,18,19], but few studies have investigated those biomarkers in tumors of salivary glands. Therefore, the present study aimed to explore the association between the expression of CD44s, CD44v6, IL-1β, CXCL1, and CXCR2, and the degree of their differentiation/aggressiveness in tumors of salivary glands.

## 2. Materials and Methods

### 2.1. Ethical Statement

The Ethics Committees for Human Research at Chiang Mai University’s Faculty of Dentistry gave their approval to the current research (no. 84/2020).

### 2.2. Patients

This experimental study included 38 samples from patients diagnosed with salivary gland tumors from 2010 to 2020 in the Faculty of Dentistry, Chiang Mai University.

All collected formalin-fixed and paraffin-embedded (FFPE) specimens were required to meet the inclusion and exclusion criteria. The inclusion criteria were: (1) specimens that had a primary tumor located in the salivary gland, (2) all specimens were classified based on the 4th edition of the World Health Organization (WHO) Classification of Head and Neck Tumors and histopathologically diagnosed as either pleomorphic adenoma, mucoepidermoid carcinoma, adenoid cystic carcinoma, or clear cell carcinoma [20], and (3) the diagnosis was confirmed by two pathologists. The exclusion criterion was that specimen with inadequately or improperly fixed tissue were excluded. Notably, the other comorbidities or risk factors of the patients were not consider as exclusion criteria.

### 2.3. Tissue Preparation for Histopathological Examination

Specimens were collected after biopsy, and all specimens were fixed in 10% paraformaldehyde for 24 h. Then, specimens were proceeded for the paraffin-embedding. The paraffin-embedded specimens were sectioned at 3 μm thickness using a microtome (M325, Thermo Fisher Scientific, Shanghai, China) and mounted on a glass slide [21]. All sections were stored at room temperature until further immunohistochemical analysis.

### 2.4. Immunohistochemistry for CD44s, CD44v6, IL-1β, CXCL1 and CXCR2

The immunohistochemical staining in each slide was performed using the IHC Select™ Immunoperoxidase Secondary Detection System (EMD Millipore Corp., Burlington, MA, USA). All slides underwent the process of deparaffinization and rehydration by using a graded series of alcohols [22]. All sections were incubated in xylene twice at 25 °C for 3 min each time. Next, the sections were incubated in a mixture of xylene and 100% ethanol (1:1) for 3 min, followed by 2 times incubation in 100% ethanol for 3 min at 25 °C. Subsequently, the sections were incubated in a graded series of alcohols, including 95%, 70%, 50%, and 30% ethanol, each for 3 min at 25 °C. Finally, the sections were incubated in distilled water for 3 min at 25 °C. Next, the sections were incubated in 10 mmol/L sodium citrate buffer (the mixture of C_6_H_5_Na_3_O_7_.H_2_O and of C_6_H_8_O_7_) pH 6.0 for 10 min at 92–95 °C for antigen retrieval [23]. After rinsing with rinse buffer twice, a thin film-like green-tinged hydrophobic barrier over the hydrated section was created by PAP pen. Then, 3% hydrogen peroxide was used to treat sections for 10 min at 25 °C. After rinsing twice with the rinse buffer, and blocked with blocking buffer for 5 min at 25 °C, each section was treated either with primary anti-CD44s, RRID:AB_2847859 (ab157107, 1:500, Abcam), anti-CD44v6, RRID:AB_726525 (ab30436, 1:500, Abcam), anti-IL-1β, RRID:AB_2804633 (no. PA5-88078, 1:500, Invitrogen), anti-CXCL1 (no. PA5-86508, 1:500, Invitrogen), or anti-CXCR2, RRID:AB_2852059 (no. PA5-102662, 1:500, Invitrogen) for 30 min at 25 °C [24]. For the negative control, sections were treated with a rinse buffer for 30 min at 25 °C. After rinsing twice with the rinse buffer, all sections were treated with 30 min of biotinylated secondary antibody, 10 min of streptavidin-HRP, and 10 min of chromogen incubation, respectively. Lastly, all sections were treated with hematoxylin for a minute, rinsed twice with the rinse buffer, and covered with a coverslip.

### 2.5. Image Acquisition and Analysis

A light microscope (Olympus CKX53, Tokyo, Japan) was used to capture a pair of images per section of all samples at 10× of magnification. The light intensity was the same for all image acquisitions.

For the image analysis, the Image J software was used to analyze the data acquisition [25,26]. Shortly, the IHC Toolbox plugin was added to the Image J software. After opening the RAW images from the Image J software, the IHC Toolbox in the Plugins was selected to access the new command box. In the new command box, the H-DAB command was selected along with the Color button, which excluded the nucleus area and left only the positive DAB color visible. Next, the threshold for the positive DAB area was adjusted. Finally, the positive DAB area was measured using the Measure command in Analyze mode. Then the data were statically tested and visualized using GraphPad Prism software.

### 2.6. Statistics

The mean and standard error of the mean for each parameter were calculated. For all multiple comparisons, data were tested for distribution using the Kolmogorov–Smirnov normality test and subsequently tested for one-way ANOVA followed by post-hoc LSD analysis. Between low- and high-grade malignant, the unpaired T-test was used to compare marker expressions. A *p* value of less than 0.05 was considered statistically significant.

## 3. Results

### 3.1. The Patients Characteristics and Salivary Gland Tumors Characteristics as Indicated by Hematoxylin and Eosin Staining

**Figure 1:**
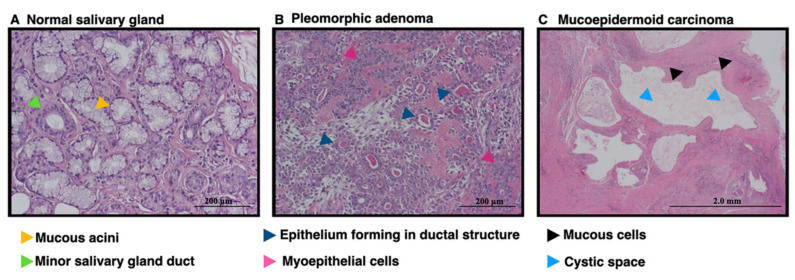
The hematoxylin and eosin (HE) staining images. (**A**–**C**) The HE staining indicated the characteristics of a normal salivary gland (bar = 200 μm), pleomorphic adenoma (bar = 200 μm), and mucoepidermoid carcinoma (bar = 2 mm).

The present study included specimens from 38 cases, including 6 specimens of normal, non-tumorous salivary glands, 13 cases of pleomorphic adenoma, 13 cases of mucoepidermoid carcinoma, 1 case of carcinoma ex pleomorphic adenoma, 3 cases of adenoid cystic carcinoma, and 2 cases of clear cell carcinoma. All specimens were obtained from various pathologic sites, including lower lip, upper lip, mandible, maxilla, palatal, retromolar pad, parotid gland, and sublingual gland. At the time of diagnosis, the age of the patients ranged from 14 to 84 years old. There were three females and three males in the non-tumorous group, ranging in age from 27 to 59 years old. The benign group included five female and eight male patients ranging in age from 20 to 62 years old. There were 11 female and 8 male cases in the malignant group. This group showed the widest age range, which was 14 to 84 years old. The demographic data is shown in Table 1. All samples were re-examined by two pathologists to confirm the histopathologic diagnoses and were divided into normal, benign, and malignant groups. The normal group, as the control, exhibited normal mucous cells and normal salivary gland ducts (Figure 1). Samples showing pleomorphic adenoma, represented in the benign group, showed epithelial components that present as duct and myoepithelial cells (Figure 1B). The malignant group which included mucoepidermoid carcinoma, demonstrated cystic spaces surrounded by mucous cells (Figure 1C). The malignant group was then subdivided into two subgroups: low-grade and high-grade malignancy [27]. The low-grade malignancy included seven low grade mucoepidermoid carcinoma cases and two clear cell carcinomas, whereas the high-grade malignancy group included one high grade mucoepidermoid carcinoma, one intermediated grade mucoepidermoid carcinoma, four intraosseous mucoepidermoid carcinoma, one carcinoma ex pleomorphic adenoma, and three adenoid cystic carcinoma cases [20,27].

### 3.2. Salivary Gland Tumors Increased the Expression of CD44s and CD44v6

**Figure 2:**
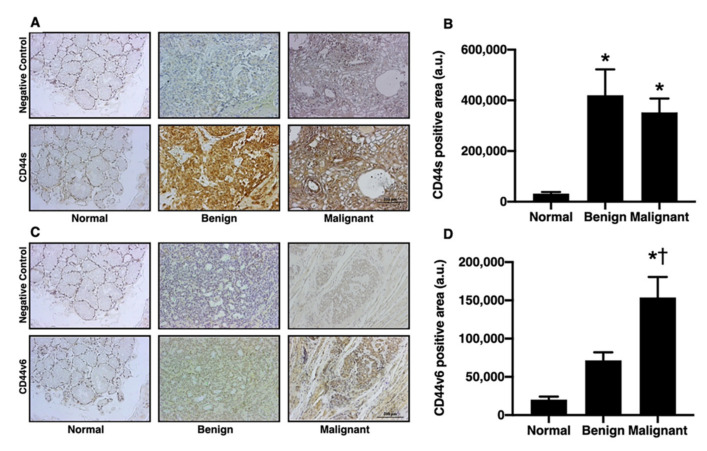
The CD44s and CD44v6 expression in oral salivary gland tumors. (**A**) The example images of the CD44s positive areas as indicated by IHC staining in normal, benign, and malignant groups (left to right panel, respectively) (bar = 200 μm). (**B**) The benign group and malignant group equally increased the CD44s positive area. (**C**) The example images of CD44v6 positive areas as indicated by IHC staining in normal, benign, and malignant groups (left to right panel, respectively) (bar = 200 μm). (**D**) CD44v6positive area increased in the benign group, while the malignant group had the highest level of CD44v6 positive area. * *p* < 0.05 vs. normal, † *p* < 0.05 vs. benign.

Several markers, including aldehyde dehydrogenase (ALDH), octamer-binding transcription factor (OCT4), and CD44, have been shown to identify tumor stem cells in various types of tumors [5,6]. The different isoforms of CD44, such as CD44s and CD44v6, have also been related to poor prognosis and metastasis in breast cancer [11]. In addition to those previous studies, the present study investigated the levels of CD44s and CD44v6 in salivary gland tumors. In comparison with the normal group, the benign and malignant groups had significantly increased CD44s positive areas (Figure 2A,B; *p* < 0.05). Furthermore, when compared to the normal group, CD44v6 expression was higher in the benign and malignant groups. Interestingly, the level of expression of CD44v6 was at the highest level in the malignant group (Figure 2C,D; *p* < 0.05). These results indicated that CD44v6 and CD44s might be biomarkers for benign and malignant salivary gland tumors, and CD44v6 could be potential biomarkers for determining the level of tumor aggression.

### 3.3. IL-1β and CXCR2, but Not CXCL1, were Significantly Increased in the Tumorous Salivary Glands in Comparison to the Non-Tumorous Glands

**Figure 3:**
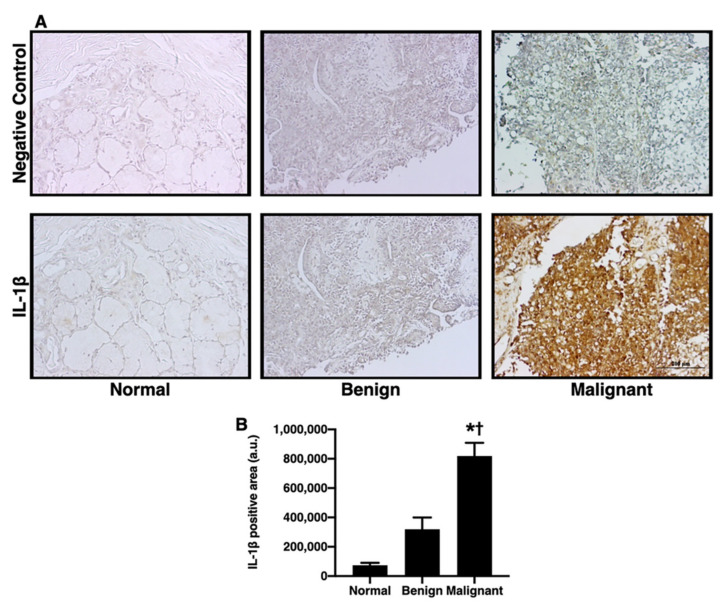
The IL-1β expression in oral salivary gland tumors. (**A**) The example images of IL-1β positive areas as indicated by IHC staining in normal, benign, and malignant groups (left to right panel, respectively) (bar= 200 μm). (**B**) Malignant group, but not benign group, increased IL-1β positive area. * *p* < 0.05 vs. normal, † *p* < 0.05 vs. benign.

**Figure 4:**
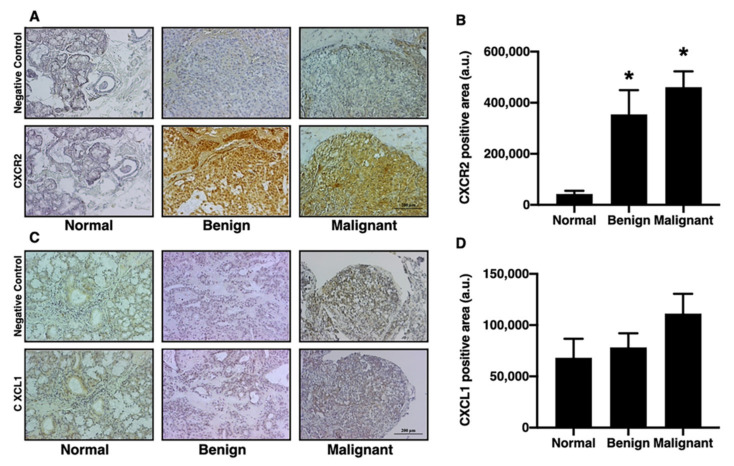
The CD44s and CD44v6 expression in oral salivary gland tumors. (**A**) The example images of CXCR2 positive area as indicated by IHC staining in normal, benign, and malignant groups (left to right panel, respectively) (bar = 200 μm). (**B**) Benign group and malignant group increased CXCR2 positive. (**C**) The example images of the CXCL1 positive area as indicated by IHC staining in normal, benign, and malignant groups (left to right panel, respectively) (bar = 200 μm). (**D**) CXCL1 positive area was no change among all groups. * *p* < 0.05 vs. Normal.

The inflammatory processes and pro-inflammatory cytokines play a crucial role in differentiation of the tumor [28]. Oral cancer has been linked to pro-inflammatory cytokines, such as CXCL1 and IL-1β [12,19,29,30,31,32,33]. Previous studies have found that the increase in IL-1β is be involved in bone metastasis [34]. The levels of IL-1β, CXCL1, and CXCR2 in salivary gland tumors were investigated in this study. When compared with the normal and benign groups, the IL-1β positive area of the malignant group was greater (Figure 3A,B; *p* < 0.05). Next, we determined the expression of CXCR2 and CXCL1. In comparison to the normal group, the CXCR2 positive area was increased to an equal extent in the benign group and malignant group (Figure 4A,B; *p* < 0.05). The CXCL1 positive area, on the other hand, did not differ between the groups (Figure 4C,D). These results indicated that IL-1β could be one of the biomarkers for determining the level of aggressiveness for salivary gland tumors, while CXCR2 expression could be used to differentiate salivary gland tumors.

### 3.4. High-Grade Malignant Tumors had Higher Levels of CD44s, IL-1, CD44v6, CXCL1, and CXCR2 Expression Than Low-Grade Malignant Tumors

**Figure 5:**
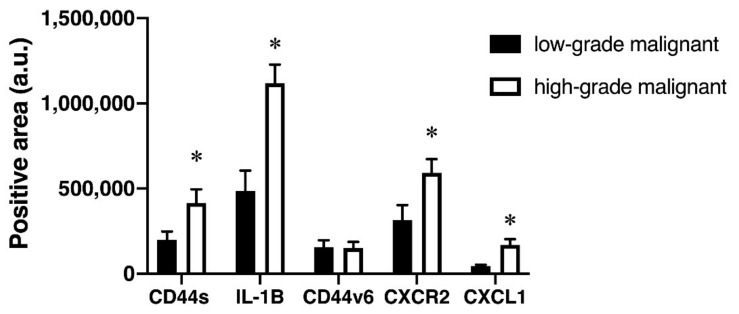
The expression of CD44s, IL-1β, CD44v6, CXCR2, and CXCL1 in different grades of malignant salivary gland tumors. * *p* < 0.05 vs. low-grade malignant salivary gland tumor.

Based on the Classification of Head and Neck Tumors by WHO, malignancy is classified into two types: low-grade malignancy and high-grade malignancy [20]. CD44 can regulate the malignant processes such as angiogenesis and tumor growth [35]. CD44 may play a role as a prognostic factor independently for low-grade gliomas [35]. A previous study showed that CD44s expression correlates with high-grade and advanced-stage ovarian carcinoma [36]. To better understand the relationship between these markers and their degree of differentiation/aggressiveness in salivary gland tumors, we further compared the expression of all markers between low- and high-grade malignant groups. The results demonstrated that the high-grade malignant group had more CD44s, IL-1β, CXCR2, and CXCL1 positive areas than the low-grade malignant group (Figure 5; *p* < 0.05). Conversely, there was no difference between the CD44v6 positive area in the low- and high-grade malignant groups (Figure 5). Overall, these findings indicate that IL-1β may play a significant role in the aggressiveness of salivary gland tumors, as it was expressed only in the malignant group as well as its expression was high in the samples of the high-grade malignant group of salivary gland tumors.

## 4. Discussion

The following are the main findings of this study: (1) in both the benign and malignant groups, CD44s expression was equally increased; (2) the CD44v6 expression gradually increased with the severity of tumors; (3) only the malignant group had an increase in IL-1β expression; (4) both the benign and malignant groups had increased CXCR2 expression, but not CXCL1; and (5) the high-grade malignant group had higher expression of CD44s, IL-1β, CXCR2, and CXCL1, but not CD44v6, than the low-grade malignant group.

CD44 is a cancer stem-like cell marker, particularly breast cancer, and CD44 expression is related to metastatic disease and therapeutic resistance [37,38]. Of the various isoforms of CD44, the two isoforms, CD44s and CD44v6, have been studied in regard to their roles as biomarkers for bladder cancers [39], gastric adenocarcinoma, and colorectal adenocarcinoma [40]. It has been shown that CD44s is highly expressed in pancreatic cancer [17], breast cancer [18], lung cancer [41], and colorectal cancer [42]. Similarly, previous studies have shown that CD44v6 is highly expressed in cases of pancreatic carcinoma [43], ovarian cancer [44], and colon cancer [45]. Furthermore, previous studies have reported the high expression of CD44s in the salivary gland tumors [46,47]. The present study also confirmed that CD44s and CD44v6 are increasingly expressed by salivary gland tumors and CD44v6 is highly expressed in malignant tumors.

The CXCR2 has been used as a marker for triple-negative breast cancer [37,48]. This study also reported that CXCR2 expression was equally increased in the benign group and malignant group. CD44s and CXCR2 expressions, on the other hand, have been shown to differ significantly between low- and high-grade malignant groups. Collectively, both CD44s and CXCR2 could be biomarkers for salivary gland tumors and an increase in CD44s and CXCR2 expression might be associated with the aggressiveness of the malignancy of salivary gland tumors. Surprisingly, the expression of CXCL1, a small peptide belonging to the CXC chemokine family, was not different between groups. This finding was not similar to the changes in its receptor, the CXCR2, suggesting that CXCL1 might not be the only ligand that can bind and activate CXCR2. Previous studies have shown that not only CXCL1, but also IL-8, can activate CXCR2 in cancerous cells [49,50].

In addition to CD44s and CXCR2, CD44v6 expression was up in both the benign and malignant groups, with the highest levels in the malignant samples. Surprisingly, we found that CD44v6 expression did not differ significantly between low-grade and high-grade malignant samples. The high CD44v6 expression was found to be associated with the malignant stage, and it is possible that this level reached the maximal level of CD44v6 expression, because we did not detect a significant difference in CD44v6 expression between low- and high-grade malignancy. In support of this finding, Tjhay and colleagues reported that a high CD44v6 expression was involved in poor prognosis of ovarian cancer [44]. Li and colleagues revealed that an increased CD44v6 expression was linked to a lower survival rate in pancreatic cancer patients [43]. Collectively, CD44v6 is a potential biomarker for malignancy in salivary glands.

For many years, researchers have investigated the links between inflammation and various forms of cancer [28,51,52,53]. It has been shown that inflammation is involved in cell proliferation, prolonged cell survival, and promotion of the progression of tumors [52,54]. IL-1β levels were found to be higher in oral cancers in previous studies [12,19,29,30,31,32,33]. In breast cancer, IL-1β was found to be involved in bone metastasis [34]. IL-1β also promotes immune suppression in pancreatic cancer [55]. Our findings also indicated that IL-1β was robustly increased in malignant salivary gland tumors. Collectively, IL-1β could be a factor linking malignancy and inflammation of salivary gland tumors. Several studies have reported a connection between cell surface markers and cancerous lesions, including CD44s, CD44v6, and CXCR2, and IL-1β [12,13,56]. A previous study showed that soluble CD44 raised the level of IL-1β in breast cancer, and IL-1β could also be activated by soluble CD44 secretion [56]. Our results also showed that not only IL1-β expression in salivary gland tumors was elevated, but also both CD44s and CD44v6 were increased. Thus, it is possible that IL-1β and CD 44 might interact with each other. In addition, an association between IL-1β and CXCR2 has been reported in oral cancers [12,13]. Therefore, our findings suggest that CD44s, CD44v6, and CXCR2 might be involved in the release of IL-1β in salivary gland tumors, and IL-1β may play a crucial role in the promotion of the aggressiveness of salivary gland tumors. A key finding from this study is that IL-1β is a potentially important biomarker for salivary gland malignancy.

The major limitation of this study was that there is no information on survival rate and clinical related symptoms considered in this study. However, the major limitations have been investigated in many previous studies, which demonstrated that CD44s was found to be associated with a lower 5-year overall survival rate in pharyngeal and laryngeal cancer [57] and poorer survival in chondrosarcoma patients [58]. Furthermore, in head and neck cancer, CD44v6 has been linked to tumor invasion and metastasis [59]. Moreover, an increase in CXCR2 expression was reported to be related with poor prognosis in acute myeloid leukemia [60] and invasion of gastric cancer [61]. CXCL1 has also been linked to a poor prognosis in patients with gastric cancer [62], HNSCC [63], glioblastoma [64], and colorectal cancer [64]. Previous studies reported that increased IL-1β level was reported to be related with bone metastasis in breast cancer [34] and promoted immune suppression in pancreatic cancer patients [55]. All these studies supported our findings that IL-1β found in the malignant group may be associated with poor prognosis. Further studies should investigate all these markers in salivary gland tumors and survival rate as well as clinically relevant symptoms. In addition, the sample size in each group was small, limiting the statistical rigor of the study and a larger number of samples should be included in a future study.

## 5. Conclusions

In salivary gland tumors, there has been an increase in the expression of CD44s, CD44v6, CXCR2, and IL-1β. Only IL-1β may play a crucial role in the aggressiveness of salivary gland tumors, because it was expressed only in the malignant group and its expression was high in the high-grade malignancy. Therefore, IL-1β could be a biomarker for investigating the aggressiveness in salivary gland tumors.

## Figures and Tables

**Table 1:** The demographic characteristics of the study population.

Groups	N	Female Subjects	Male Subjects	Mean Age ± SD (Range) (Years)	Locations								
					LL	UL	MD	MX	Cheek	P	RP	PG	SL
Normal group	6	3	3	39.0 ± 12.45 (27–59)	1		1		1	1		1	1
Benign group	13	5	8	42.23 ± 18.39 (20–62)		2		1	1	9			
Malignant group	19	11	8	52.63 ± 14.60 (14–84)			4	2		2	1		
Total	38	19	19	46.72 ± 16.95 (14–84)	1	2	5	3	2	12	1	1	1

LL, lower lip; UL, upper lip; MD, mandible; MX, maxilla; P, palatal; RP, retromolar pad; PG, parotid gland; SL, sublingual gland.

## Data Availability

Not applicable.
